# ERPs in an oddball task under vection-inducing visual stimulation

**DOI:** 10.1007/s00221-016-4748-8

**Published:** 2016-08-03

**Authors:** Paweł Stróżak, Piotr Francuz, Paweł Augustynowicz, Marta Ratomska, Agnieszka Fudali-Czyż, Bibianna Bałaj

**Affiliations:** 1 Department of Experimental Psychology, The John Paul II Catholic University of Lublin, Al. Racławickie 14, 20-950 Lublin, Poland; 2 Faculty of Economics, Maria Curie Skłodowska University in Lublin, Plac Marii Curie-Skłodowskiej 5, 20-031 Lublin, Poland; 3 Faculty of Humanities, Nicolaus Copernicus University in Toruń, ul. Gagarina 11, 87-100 Toruń, Poland

**Keywords:** ERPs, Vection illusion, P1, N2, P3

## Abstract

The neural mechanisms underlying the vection illusion are not fully understood. A few studies have analyzed visually evoked potentials or event-related potentials (ERPs) when participants were exposed to vection-inducing stimulation. However, none of them tested how such stimulation influences the brain activity during performance of the simultaneous visual task. In the present study, ERPs were recorded while subjects (*N* = 19) performed a discrimination oddball task. Two stimuli (O or X) were presented on the background of central and peripheral visual fields consisting of altered black and white vertical stripes that were stationary or moving horizontally. Three different combinations of these fields were created: (1) both center and periphery stationary (control condition), (2) both center and periphery moving, (3) center stationary and periphery moving. Mean reaction times to targets were shortest in the control condition. The amplitudes of P1 and N2 at occipital locations, and the amplitude of P3 at frontal, central, and parietal locations, were attenuated, and the P3 exhibited longer peak latency when both central and peripheral visual fields were moving. These potentials reflect initial sensory processing and the degree of attention required for processing visual stimuli and performing the task. Our findings suggest that the integration of central and peripheral moving visual fields enhances the vection illusion and slows down reaction times to targets in the oddball task and disrupts the magnitude of electrophysiological responses to targets.

## Introduction

When we move around the environment, signals from visual, vestibular, and proprioceptive sensory systems are integrated in order to construct the perception of self-motion. Sometimes, however, systematic motion of visual environment can induce the illusion of self-motion in a stationary observer (Dichgans and Brandt 1978; Palmisano et al. 2015). This sensation is called vection and can frequently occur in motion simulators, virtual three-dimensional environments, or in reality, e.g., while one is seated on a stationary train and another train moves on the adjacent track.

Studies of vection illusion have primarily focused on perceptual aspects of this phenomenon (Johansson 1977; Lee 1980; Koenderink 1986; Lappe et al. 1999). Individual differences in the duration and the subjective strength of vection, as well as the effects of practice, were also highlighted (Kennedy et al. 1995). Several further studies have analyzed the neural correlates of vection. Positron emission tomography (PET) showed that visual motion stimulation inducing circular vection activates parieto-occipital visual areas and simultaneously deactivates the parieto-insular vestibular cortex (Brandt et al. 1998). This pattern of results indicated that the illusion of self-motion is managed by an inhibitory interaction between the visual and the vestibular systems. Consistent with this functional interpretation of vection, the results of subsequent PET study showed similar parieto-occipital activations and retroinsular deactivations during rollvection and linearvection (Deutschländer et al. 2004), although varied activations in other cortical areas between these two kinds of vection were also found in that study. In another PET study, it has also been demonstrated that the illusion of self-motion is associated with the activation of limbic structures (Beer et al. 2002). Also, studies using functional magnetic resonance imaging (fMRI) identified neural correlates of illusory self-motion, ranging from early motion-sensitive visual areas to higher-order regions involved in visual imagery and decision making (Kleinschmidt et al. 2002; Kovács et al. 2008).

There is a relative scarcity of studies on vection using EEG-based (electroencephalography) techniques. EEG is a promising technique that might be applied in vection research, particularly because of its high temporal resolution (Keshavarz et al. 2015). Such studies focused on topographic (Tokumaru et al. 1999) or spatiotemporal (Wiest et al. 2001) signal distributions during vection. Thilo et al. (2003) used visually evoked potentials and found reduced N70 amplitude in response to pattern reversals in the central visual field when subjects experienced self-motion compared with the experience of object motion. This finding reflects the early visual cortex deactivation when a visual flow elicits vection, and supports the results of fMRI study (Kleinschmidt et al. 2002). Keshavarz and Berti (2014) measured event-related potentials (ERPs) on a horizontally moving pattern of altered black and white vertical stripes. This pattern was divided into a central field and a peripheral field. The N2 amplitude at occipital sites was decreased when the central field was moving and the peripheral field was stationary, the condition in which the vection was the weakest. On the other hand, the largest N2 amplitude at the same sites was recorded when the central field was stationary and the peripheral field was moving, which was associated with the strongest feeling of vection. Because ERPs were recorded only during initial processing of visual motion, Keshavarz and Berti (2014) suggested that the N2 potential might reflect the integration of peripherally and centrally presented visual information, which precedes the actual perception of vection.

Although studies by Thilo et al. (2003) and Keshavarz and Berti (2014) provide valuable insight into how different stages of vection are reflected in the amplitudes of visually evoked and event-related potentials, none of them tested the impact of vection-inducing visual stimulation on the simultaneous activity performed by the subjects. Thus, the goal of the present study is to assess whether different patterns of visual stimulation inducing vection affect the performance of the additional task. We utilized a simple oddball visual paradigm in which subjects had to respond to infrequent targets (deviants) presented among frequent standard stimuli. Also, we were interested in whether ERPs associated with this additional task would be influenced by the vection-inducing stimulation. In order to induce the vection illusion, we used the moving patterns of vertical stripes originally devised by Keshavarz and Berti (2014). However, we superimposed them on the continuous presentation of standard and deviant stimuli in the oddball task.

We suggest that vection-inducing stimulation should affect a secondary visual task because such stimulation constitutes higher perceptual load than stimulation not inducing vection. Perceptual load is defined as the number of different-identity items that need to be perceived (Lavie 2005). The movement of central and peripheral visual fields can be regarded as one of such items and, in comparison with stationary visual fields, processing this movement would consume additional attentional resources. As a result, it would diminish neural responses to stimuli in the simultaneous visual task, as well as decrease the level of behavioral performance of this task. This is consistent with the perceptual load theory, according to which under conditions of high perceptual load attentional resources are used to process stimuli relevant to the main task and no (or few) resources are left for any additional task (Lavie 2005). This is also consistent with a broader theoretical framework of attentional resources (Kahneman 1973; Marois and Ivanoff 2005), as well as with previous studies in which the sensation of vection resulted in reduced brain activity (Brandt et al. 1998; Kleinschmidt et al. 2002; Thilo et al. 2003).

Thus, we hypothesized that the amplitudes of ERPs to targets would be attenuated during the stimulation inducing vection (both center and periphery moving, and, to a lesser extent, center stationary and periphery moving) compared with control stimulation not inducing vection (both center and periphery stationary). We also hypothesized that the peak latencies of ERPs would be longer under the vection-inducing stimulation than under the stimulation not inducing vection. Similarly to the case of ERP amplitudes, longer peak latencies are regarded as indicators of the influence of attenuation factors on the course of cognitive processes reflected by ERPs. We also intended to analyze the behavioral results. We hypothesized that during the stimulation inducing vection (compared with the control condition) the subjectively reported sensations of vection would be more frequent, the accuracy of responses in the oddball task would be lower, and reaction times to targets in that task would be longer.

It is also worth noting that the underlying assumption of our hypotheses is that vection is not merely an epiphenomenon of the brain activity, but has behavioral relevance and helps to update internal representations of our position and orientation in the environment (Palmisano et al. 2015). However, this does not come without a cost, which is reflected by higher perceptual load under vection-inducing stimulation and the need for more attentional resources to process it.

## Methods

### Participants

Twenty-five healthy volunteers participated in the experiment. EEG data were analyzed for nineteen subjects (*N* = 19, 11 female) for whom a sufficient number of artifact-free EEG epochs was provided (mean age *M* = 21.37, SD = 1.86, range = 20–26). Behavioral data were analyzed for eighteen subjects (*N* = 18, 10 female) because behavioral data from one participant (female) were lost because of a software malfunction (EEG data from this participant were preserved). All subjects had vision that was normal or corrected to normal and no history of neurological disorders. Participants gave written consent prior to the experiment. Ethical Committee of the University Institute of Psychology approved the procedures.

### Stimuli and procedure

The stimulus pattern used in the experiment consisted of a pattern of altered black and white vertical stripes divided into a central field and a peripheral field. Both fields were stationary or were moving horizontally. We created one control and two experimental patterns. In control conditions, both center and periphery stripes were stationary (CS + PS). In experimental conditions, both center and periphery stripes were moving (CM + PM) or center stripes were stationary and periphery stripes were moving (CS + PM). In the first experimental condition, both fields were moving in the opposite direction at the same speed. The number of trials within this condition was counterbalanced according to the movement of both fields on the left or on the right. The same was true for the movement of the periphery in the second experimental condition. We abandoned the condition in which the center was moving and the periphery was stationary, as this produces the weakest sensation of vection (Keshavarz and Berti 2014). Instead, we added the control condition in which both fields were stationary in order to provide the basic stimulation not inducing vection. This condition was absent in the design by Keshavarz and Berti (2014).

The stimulus pattern described above was superimposed on the continuous presentation of standard (“O”) and deviant (“X”) stimuli as in the typical oddball task. The standards were frequent (75 %) images and the deviants (targets) infrequent (25 %) images. Both were presented in the center of the central field consisting of vertical stripes. Each stimulus was on screen for 100 ms with an interstimulus interval of random duration in the range of 2000–3000 ms. The fixation cross was on screen whenever the stimulus was not. Subjects were instructed to focus on the fixation cross throughout the whole experiment. Subjects pressed the key any time they observed a deviant stimulus. Additionally, subjects pressed another key whenever they felt the sensation of vection. This was done in order to obtain subjective, self-reported indications of vection.

Before the start of the experimental session, the definition of vection was introduced to subjects. The phenomenon of the train illusion was described for each participant as a good illustration of vection. Subjects were instructed that whenever they feel the subjective movement of their own bodies they should press and immediately release the specified button. Additional symptoms that can accompany the perception of vection (motion sickness, dizziness) were also delineated to allow for a better understanding and self-awareness of vection. A short practice session was administered before the experimental session, incorporating one control (CS + PS) and two experimental (CS + PM, CM + PM) patterns. After completing the practice session, subjects were asked whether they understand how to perform the task and how to respond when they feel the sensation of vection. None of the subjects queried the instructions given.

Some 1280 trials in total (960 standards and 320 targets), divided into eight blocks, were presented in the experimental session. Within each block 120 standards and 40 targets were presented, counterbalanced for all conditions. The control pattern (CS + PS) was presented between each motion pattern (CM + PM or CS + PM), as well as at the beginning and at the end of each block. Thus, the number of trials with control patterns was twice as many as the CM + PM and CS + PM patterns. The number of trials with CM + PM and CS + PM patterns was equal. Each motion pattern stimulation lasted 45 s. There were four CS + PS patterns, two CM + PM patterns, and two CS + PM patterns within each block. Thus, each block lasted 8 min. A short break (approximately 3 min) was provided between each block.

Stimuli were presented on a 20-inch LCD computer monitor with a display resolution of 1680 × 1050 pixels and refresh rate of 60 Hz. Subjects were seated at a viewing distance of 30 cm from the monitor. The central field of the stimulus pattern measured 25.9 cm horizontally and 16.2 cm vertically, which subtended approximate visual angles of 45° horizontally and 30° vertically. The peripheral field measured 51.8 cm horizontally and 32.4 cm vertically (field of view 96° × 60°).

### EEG recording and analysis

An electroencephalogram (EEG) was recorded continuously with 64 active electrodes (ActiCAP, Brain Products, Munich, Germany) connected to a high-input-impedance amplifier (200 MΩ, GES 300, Electrical Geodesics, Inc., Eugene, OR). The EEG was referenced to an FCz electrode and digitized at a sampling rate of 250 Hz. Electrode impedances were kept below 10 kΩ. Offline, a digital band-pass filter (0.1–40 Hz) was used and the EEG was re-referenced to linked mastoids. Eye movements were corrected by independent component analysis (Delorme et al. 2007). Remaining artifacts were rejected using the moving window peak-to-peak amplitude method with window width 200 ms, window step 100 ms, and threshold ±100 µV (Luck 2014). Epochs were created, beginning at 200 ms prior to stimulus onset and ending 600 ms after it. ERPs were baseline-corrected relative to the pre-stimulus interval. Six participants were removed from the analyses because of apparatus malfunction (two subjects) or a strongly distorted EEG signal resulting in a low number of artifact-free EEG epochs (four subjects). Grand averages were computed only for targets, separately for three motion pattern conditions (CS + PS, CM + PM, CS + PM). The mean numbers of trials per subject per condition used to calculate ERPs were as follows: CS + PS condition (*M* = 146.84, SD = 12.77, range = 109–160), CM + PM condition (*M* = 73, SD = 5.07, range = 63–79), and CS + PM condition (*M* = 72.63, SD = 6.17, range = 57–79).

## Results

### Behavioral results

Three repeated measures one-way ANOVAs including the motion pattern condition as a factor (CS + PS, CM + PM, CS + PM) were conducted separately for mean proportion of correct responses to targets, mean reaction times of correct responses to targets, and mean proportion of subjectively felt sensations of vection (although it is scarcely possible for the control condition to produce more frequent sensations of vection than experimental conditions, we decided to include this condition in all behavioral analyses for brevity and completeness). These measures are given in Table 1.

**Table 1:** Means (*M*) and standard deviations (SD) for accuracy (mean proportion of correct responses to targets), reaction times in milliseconds (mean reaction times of correct responses to targets), and vection frequency (mean proportion of subjectively felt sensations of vection) in different motion pattern conditions

	Motion pattern					
	CS + PS		CM + PM		CS + PM	
	*M*	SD	*M*	SD	*M*	SD
Accuracy	.98	.04	.99	.02	.98	.04
Reaction times (ms)	471.92	67.01	484.58	59.66	477.51	70.18
Vection frequency	.04	.12	.30	.39	.22	.31

CS + PS, center and periphery stationary; CM + PM, center and periphery moving; CS + PM, center stationary and periphery moving

No significant effect of motion pattern on mean proportion of correct responses to targets occurred (Greenhouse-Geisser corrected), [*F*(1.33, 22.58) = 0.87, *p* = 0.391]. There were no differences between CS + PS (*M* = 0.98, SD = 0.04), CM + PM (*M* = 0.99, SD = 0.02), and CS + PM (*M* = 0.98, SD = 0.04) conditions.

A significant effect of motion pattern on mean reaction times of correct responses to targets was found [*F*(2, 34) = 6.42, *p* < 0.01, *η* ^2^ = 0.27]. The shortest reaction time of correct responses to targets was in the CS + PS condition (*M* = 471.92 ms, SD = 67.01). Planned comparisons revealed, as hypothesized, significant differences between CS + PS and CM + PM conditions (*M* = 484.58 ms, SD = 59.66; *p* < 0.01). Mean reaction times of correct responses to targets in the CS + PM condition (*M* = 477.51 ms, SD = 70.18) did not differ significantly when compared with CS + PS condition, although the significance level was only slightly above the *p* < .05 threshold (*p* = 0.056). The difference between CS + PM and CM + PM conditions was also nonsignificant (*p* = 0.113).

Significant effect of motion pattern on mean proportion of subjectively felt sensation of vection was found [*F*(2, 34) = 3.54, *p* < 0.05, *η* ^2^ = 0.17], indicating the least frequent vection ratings in the CS + PS condition (*M* = 0.04, SD = 0.12). Planned comparisons revealed, as hypothesized, significant differences for the CS + PS condition compared with the CM + PM condition (*M* = 0.30, SD = 0.39; *p* = 0.019) and with the CS + PM condition (*M* = 0.22, SD = 0.31; *p* = 0.025). There was no difference between CM + PM and CS + PM conditions (*p* = .503).

It has to be noted that the mean proportions of self-reported vection sensations were rather modest. Thus, it is hard to determine whether the above-mentioned differences in reaction times to targets between CS + PS and CM + PM conditions reflected the influence of vection per se, or were rather the effect of general visual stimulation. In order to address this issue, we performed the additional analysis on reaction times to targets in an oddball task, constraining ourselves solely to the reactions during both motion patterns (CS + PM and CM + PM). We separated the response times into two phases: before and after subjects’ button presses indicating subjectively felt sensation of vection within each motion pattern stimulation that lasted 45 s. We then averaged reaction times to targets preceding the button presses (before vection) and reaction times to targets succeeding the button presses (after vection). We excluded reaction times from motion patterns in which there was more than one button press indicating vection, as it would be hard to classify targets into “before vection” and “after vection” conditions. We hypothesized that reaction times to targets after vection would be slower than reaction times to targets before vection, thus reflecting the factual influence of vection. This hypothesis was confirmed by the paired *t* test: *t*(41) = 1.73; *p* < .05; *d* = .27 (one-tailed). Reaction times to targets after vection (*M* = 544.05 ms, SD = 111.93) were significantly slower than reaction times to targets before vection (*M* = 521.74 ms, SD = 100.19).

### ERP results

#### ERP amplitudes

Mean ERP amplitudes to targets were taken from 120- to 160-, 170- to 200-, and 360- to 500-ms time windows to capture P1, N2, and P3 components, respectively. For P1 and N2 components, we restricted our analyses to occipital channels (O1, Oz, O2), similarly to Keshavarz and Berti (2014). We conducted two repeated measures ANOVAs with motion pattern condition (CS + PS, CM + PM, CS + PM) and laterality (left/middle/right) as factors, separately for P1 and N2. For the P3 component, we focused on frontal (F3, Fz, F4), central (C3, Cz, C4), and parietal channels (P3, Pz, P4), as this potential typically shows broad topographic distribution over the scalp. For the amplitude of this potential, we conducted three-way repeated measures ANOVA with motion pattern condition (CS + PS, CM + PM, CS + PM), electrode site (frontal/central/parietal), and laterality (left/middle/right) as factors. The effects of laterality and electrode site are reported only when they interact with the motion pattern condition. Greenhouse-Geisser correction was applied when appropriate.

**Fig. 1:**
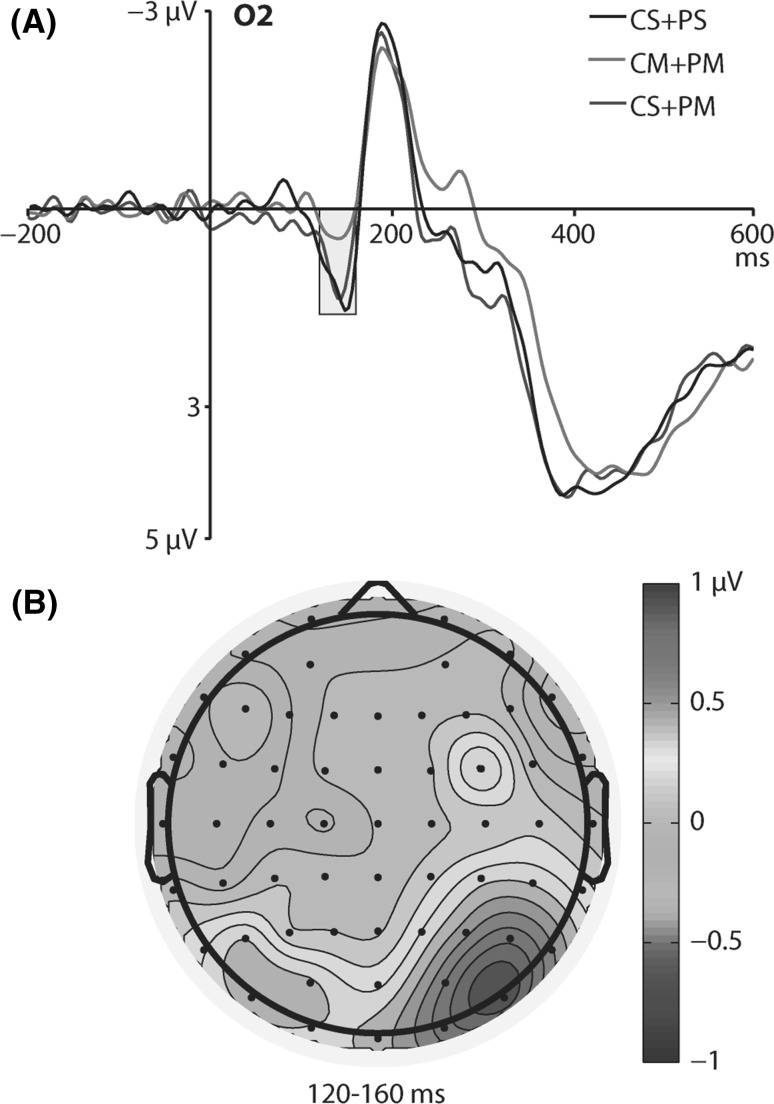
**a** ERP waveforms to targets for different motion pattern conditions at the O2 electrode (CS + PS, center and periphery stationary; CM + PM, center and periphery moving; CS + PM, center stationary and periphery moving). The P1 time window (120–160 ms) is highlighted. **b** Topographic map of the difference wave calculated by subtracting CM + PM condition from CS + PS condition in the 120- to 160-ms time window

For the P1 component, the main effect of motion pattern condition was nonsignificant [*F*(2, 36) = 2.98, *p* = 0.064], but a significant interaction between motion pattern and laterality was found [*F*(2.75, 49.48) = 3.15, *p* = 0.037, *η* ^2^ = 0.15]. The interaction was decomposed by conducting Bonferroni-corrected post hoc comparisons. They revealed that the P1 amplitude to targets was larger when center and periphery were both stationary (CS + PS; *M* = 1.1 µV, SE = 0.27) than when center and periphery were both moving (CM + PM; *M* = 0.32 µV, SE = 0.16), but only over the occipital region in the right hemisphere (at the O2 electrode; *p* = 0.035). Figure 1 depicts ERP waveforms to targets at the O2 electrode for different motion patterns (A) and the topographic map of the difference wave between CS + PS and CM + PM conditions (B).

**Fig. 2:**
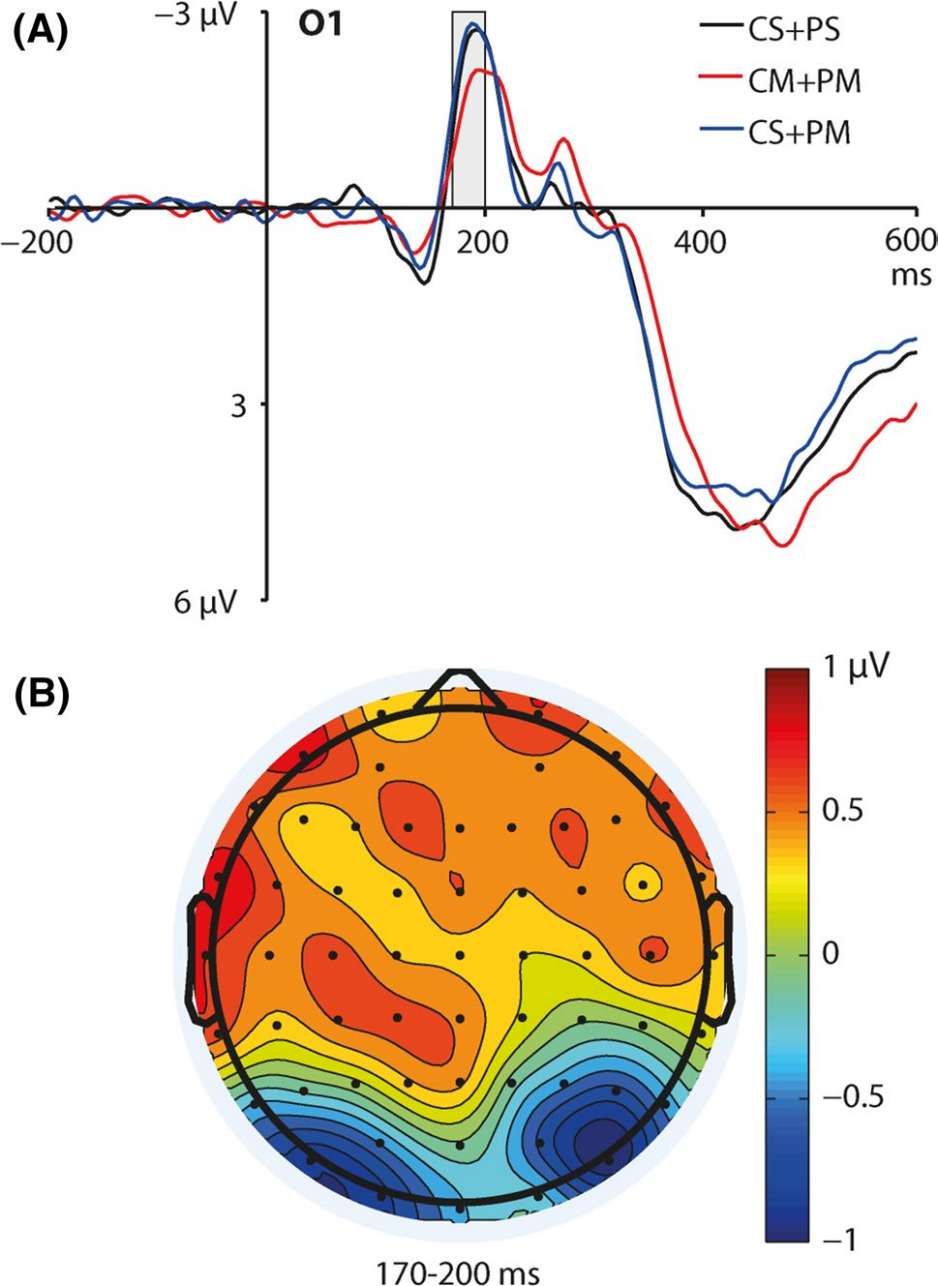
**a** ERP waveforms to targets for different motion pattern conditions at the O1 electrode (CS + PS, center and periphery stationary; CM + PM, center and periphery moving; CS + PM, center stationary and periphery moving). The N2 time window (170–200 ms) is highlighted. **b** Topographic map of the difference wave calculated by subtracting CM + PM condition from CS + PM condition in the 170- to 200-ms time window

No main effect of motion pattern condition on amplitude of the N2 component was found [*F*(2, 36) = 1.18, *p* = 0.32]. However, there was a significant interaction between motion pattern and laterality [*F*(4, 72) = 4.11, *p* = 0.005, *η* ^2^ = 0.19]. Bonferroni-corrected post hoc comparisons revealed that the N2 amplitude to targets was larger when center was stationary and periphery was moving (CS + PM; *M* = −2.38 µV, SE = 0.35) than when center and periphery were both moving (CM + PM; *M* = −1.63 µV, SE = 0.27), but only in the left hemisphere (at the O1 electrode; *p* = 0.048). Figure 2 depicts ERP waveforms to targets at the O1 electrode for different motion patterns (A) and the topographic map of the difference wave between CS + PM and CM + PM conditions (B).

**Fig. 3:**
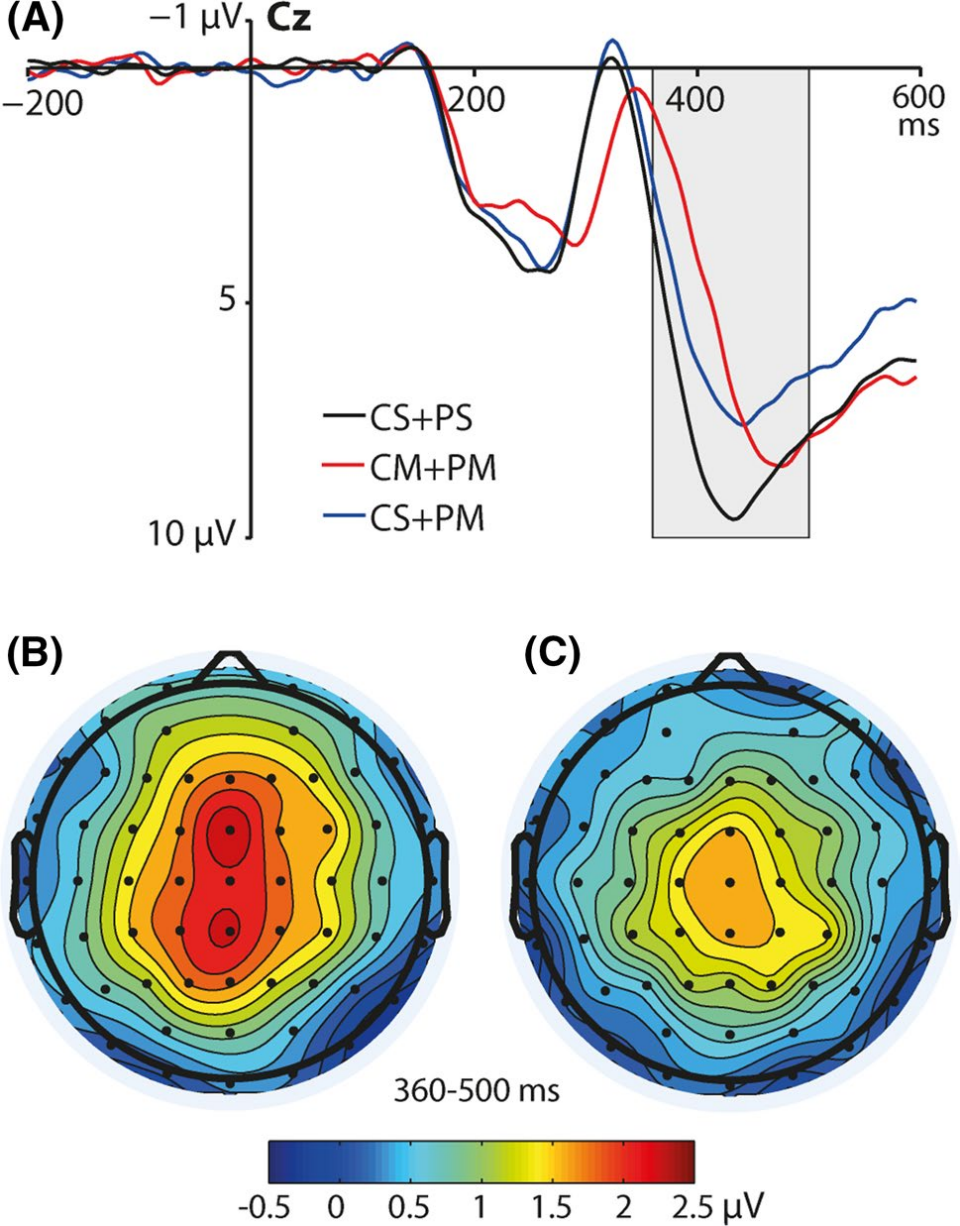
**a** ERP waveforms to targets for different motion pattern conditions at the Cz electrode (CS + PS, center and periphery stationary; CM + PM, center and periphery moving; CS + PM, center stationary and periphery moving). The P3 time window (360–500 ms) is highlighted. **b** Topographic map of the difference wave calculated by subtracting CM + PM condition from CS + PS condition in the 360- to 500-ms time window. **c** Topographic map of the difference wave calculated by subtracting CS + PM condition from CS + PS condition in the 360- to 500-ms time window

For the P3 component, we found a significant main effect of motion pattern condition [*F*(2, 36) = 10.36, *p* < 0.001, *η* ^2^ = 0.37] and a significant interaction between motion pattern and laterality [*F*(4, 72) = 6.17, *p* < 0.001, *η* ^2^ = 0.26]. For the main effect of motion pattern condition, Bonferroni-corrected post hoc comparisons revealed that the P3 amplitude to targets was larger when center and periphery were both stationary (CS + PS; *M* = 6.38 µV, SE = 0.71) than when center and periphery were both moving (CM + PM; *M* = 4.85 µV, SE = 0.53; *p* = 0.005). Also, the P3 amplitude to targets was larger in the CS + PS condition than in the center stationary and periphery moving condition (CS + PM; *M* = 5.4 µV, SE = 0.61; *p* = 0.019). There was no difference between CM + PM and CS + PM conditions (*p* = 0.193). For the interaction between motion pattern and laterality, Bonferroni-corrected post hoc comparisons revealed that the largest differences between CS + PS and CM + PM were found in the middle of the scalp (*p* = 0.001, Cohen’s *d* = 1.05), and were relatively smaller in the left hemisphere (*p* = 0.01, *d* = 0.77), and in the right hemisphere (*p* = 0.026, *d* = 0.68). That was also the case for differences between CS + PS and CS + PM conditions (*p* = 0.006, *d* = 0.83; *p* = 0.041, *d* = 0.63; *p* = 0.059, *d* = 0.59 for the middle, the left hemisphere, and the right hemisphere, respectively). Figure 3 depicts ERP waveforms to targets at the Cz electrode for different motion patterns (A), the topographic map of the difference wave between CS + PS and CM + PM conditions (B), and the topographic map of the difference wave between CS + PS and CS + PM conditions (C).

#### ERP latencies

We next sought to determine whether the motion pattern condition influenced the peak latencies of P1, N2, and P3 components to targets. As regards the ERP amplitude analyses, we focused on occipital channels for P1 and N2 components, and on frontal, central, and parietal channels for the P3 component. Mean peak latencies for P1 and N2 were submitted to separate two-way repeated measures ANOVAs with motion pattern condition (CS + PS, CM + PM, CS + PM) and laterality (left/middle/right). Mean peak latencies for P3 were submitted to three-way repeated measures ANOVA with motion pattern condition (CS + PS, CM + PM, CS + PM), electrode site (frontal/central/parietal), and laterality (left/middle/right). The effects of laterality and electrode site are reported only when they interact with the motion pattern condition. Greenhouse-Geisser correction is applied when appropriate.

No main effect of motion pattern condition [*F*(1.39, 24.96) = 0.19, *p* = 0.75], nor interaction between motion pattern and electrode site [*F*(2.68, 48.2) = 0.62, *p* = 0.59] on the peak latency of the P1 component was found. Also, no such effects were found for the N2 component [*F*(2, 36) = 0.32, *p* = 0.73 for the main effect of motion pattern condition; *F*(4,72) = 1.12, *p* = 0.35 for the interaction between motion pattern and electrode site].

For the P3 component, we found a significant main effect of motion pattern condition [*F*(2, 36) = 28.16, *p* < .001, *η* ^2^ = 0.61]. Bonferroni-corrected post hoc comparisons revealed that the P3 latency to targets was shorter when center and periphery were both stationary (CS + PS; *M* = 429.31 ms, SE = 5.62) than when center and periphery were both moving (CM + PM; *M* = 458.95 ms, SE = 5.22; *p* < .001). Also, P3 latency was shorter in the center stationary and periphery moving condition (CS + PM; *M* = 436.19 ms, SE = 6.38) than in the CM + PM condition (*p* < .001). There was no difference between CS + PS and CS + PM conditions (*p* = 0.343).

## Discussion

The analysis of behavioral results revealed that participants reported the feeling of vection most frequently in the CM + PM and CS + PM conditions. During the CS + PS condition, the sensation of vection was almost absent. This had no impact on the accuracy of responses in the oddball task, which was comparable (and very high) in each condition. However, motion pattern conditions exerted an influence on the reaction times of correct responses to targets, which were shorter in the CS + PS condition than in the CM + PM condition. Thus, performing the oddball task on the background of moving visual fields resulted in slower reaction times to targets as compared with performing the oddball task on the background of stationary visual fields.

In the first step of ERP analysis, we found the P1 component in the 120- to 160-ms time window. Its amplitude at the O2 electrode was larger when both central and peripheral visual fields were stationary (CS + PS) than when both central and peripheral fields were moving (CM + PM). It is noteworthy that Keshavarz and Berti (2014) found larger P1 amplitude in the CPOD condition (center and periphery moving in opposite directions) than in the CS + PM condition (center stationary and periphery moving). Here, we provide the evidence that the P1 amplitude is attenuated (not enhanced) when both visual fields are moving. This is probably because we recorded ERPs in response to targets presented under different motion patterns, whereas Keshavarz and Berti (2014) analyzed ERPs time-locked to the moment of changing the motion patterns. Therefore, we captured the P1 response to targets in a selective attention task in which attended stimuli typically elicit larger P1 potentials than unattended stimuli (Hillyard et al. 1998). The P1 effect was found only over the occipital areas of the scalp in the right hemisphere (at the O2 electrode; see the topographic map at the panel B of Fig. 1). This strengthens the interpretation of this effect in terms of the influence of vection-inducing stimulation in the early stages of visual processing. This is because the right hemisphere is considered to specialize in visuospatial processing (Ng et al. 2000). However, it is important not to overstate our findings toward the claim that the right-lateralized P1 effect that was found in our study can be regarded as an ERP correlate of vection. Rather, the P1 component is a correlate of early visual processing of attended stimuli and is sensitive to general optic flow intended to induce vection, but cannot be treated as a correlate of vection itself. There are two reasons that strengthen this claim. First, we haven’t found correlation between P1 amplitude and behavioral responses of vection frequency, which would be expected if P1 were preferentially linked to vection. Second, the P1 was found over the occipital areas, whereas it has been shown that it is the parietal lobe that is strongly involved in the perception of self-motion (Kovács et al. 2008).

The interpretation of the P1 potential is that it reflects a modulation of the initial sensory processing and that this processing is suppressed for unattended stimuli to reduce interference (Luck and Kappenman 2012). The pattern of results obtained in our study confirms this interpretation, as the P1 amplitude was larger in the CS + PS condition than in the CM + PM, and the immobility of both visual fields should produce less interference with the simultaneous visual task than the movement of these fields. Keshavarz and Berti (2014) found larger P1 amplitude when both visual fields were moving than when the central field was stationary and only peripheral bars were moving. They recorded ERPs in response to changes between different motion patterns and interpreted this finding as reflecting the detection of incoherent motion. However, they did not provide comparisons with the P1 amplitude recorded when both visual fields were stationary, which makes it impossible to make direct comparisons with the results of our study.

In the subsequent 170- to 200-ms time window, we found the N2 component. Similarly to Keshavarz and Berti (2014), we found that the N2 amplitude at the O1 electrode was largest when the central visual field was stationary and peripheral bars were moving (CS + PM). However, this was evident only when the N2 amplitude in the CS + PM condition was compared with the condition when center and periphery were both moving (CM + PM), but not when center and periphery were both stationary (CS + PS). Thus, it seems that the additional movement of central visual field decreased the N2 amplitude (as evident in the significant CS + PM vs. CM + PM comparison), but not the additional movement of peripheral visual field (as evident in the nonsignificant CS + PM and CS + PS conditions comparison). This is consistent with the pattern of behavioral results found in our study, such that reaction times to targets slow down significantly only when both central and peripheral visual fields are moving, but not when only periphery is moving (although it has to be noted that the CS + PM vs. CS + PS comparison for reaction times yielded the significance value only slightly above the *p* < .05 threshold).

The evident lack of difference in the N2 amplitude between CS + PS and CS + PM conditions is different from the pattern of results obtained by Keshavarz and Berti (2014). They argue that the N2 in their study was primarily affected by motion in the visual periphery and that it reflects the process of integration of peripheral motion information into the processing of the centrally presented visual information (Keshavarz and Berti 2014, p. 134). The reason for this is that Keshavarz and Berti (2014) measured ERPs solely in response to changes between different motion patterns, and the N2 potential recorded in their study reflected the initial processing of visual motion. On the other hand, we analyzed ERPs in response to targets in the oddball visual task embedded in different motion pattern stimulation. Thus, we provided evidence that the specific motion pattern with both central and peripheral visual fields in movement disrupts the magnitude of N2 response to infrequent stimuli. This is consistent with the typical interpretation of the posterior N2 component as reflecting the level of attention required for processing visual stimuli (Suwazono et al. 2000; Folstein and Van Petten 2008). This is also consistent with the above-mentioned interpretation of the P1 component in terms of the influence of visual stimulation that is intended to produce vection, but not in terms of the direct influence of vection itself.

The significant differences in the N2 domain were found only over the occipital areas of the scalp in the left hemisphere (at the O1 electrode). However, this was owed to the array of electrodes submitted to statistical analyses. As can be seen in panel B in Fig. 2, the distribution of the N2 potential over occipital areas was bilateral. Thus, it seems that at the later stage of processing (as compared with the earlier, right-lateralized P1 potential), the influence of vection-inducing stimulation is not restricted to the right hemisphere. This is consistent with the functional interpretation of N2, which reflects attentional rather than sensory processing, and is equivalent over the left and right hemispheres (Luck 2012).

In the last 360- to 500-ms time window, we found the P3 component. In contrast to P1 and N2 potentials, which were restricted to occipital electrodes, the P3 was found in broad areas of the scalp spanning frontal, central, and parietal electrodes, with the maximum across the midline electrode sites. Such broad P3 topography is typically observed in the classic visual oddball tasks (Comerchero and Polich 1999). In our study, the P3 amplitude to targets was larger in the CS + PS than in the CM + PM and CS + PM conditions and did not differ between the CM + PM and CS + PM conditions. This pattern of results provides evidence that any movement in the background of the simultaneous visual task decreases the P3 amplitude. Furthermore, the role of the eccentricity of this movement (i.e., whether it is the movement of central or peripheral visual field) is not significant.

The analysis of ERP latencies revealed that the P3 peak latency to targets was shortest in the CS + PS and CS + PM conditions and longest in the CM + PM condition. Thus, it seems that the P3 amplitude is sensitive to any movement of the background visual fields, but the P3 latency is modulated only when both visual fields are moving. These results are consistent with the functional role of the P3 that is regarded as one of the neural measures of the degree of focal attention required for performing the task (Polich 2007). The results of our study suggest that additional movement of visual fields interrupts the processing operations employed in the discrimination of targets. The differences in the P3 amplitude and latency were consistent with the behavioral pattern of results, in which the shortest reaction times of correct responses to targets were found in the CS + PS condition. Thus, the larger and faster electrophysiological response to targets was recorded when both visual fields were stationary; the faster overt behavioral response to targets was detected.

Altogether, our study demonstrates the influence of vection-inducing stimulation on the performance of the simultaneous visual task and on the event-related potentials recorded during this task. We found that stimulus patterns eliciting vection decreased the amplitudes of P1, N2, and P3 event-related potentials to targets in the oddball task as compared with the stimulus pattern not inducing vection. The time course of these ERP effects reflects the time course of cognitive operations underlying the discrimination of targets, namely the processes of initial sensory processing that are susceptible to interference (right-lateralized P1), the degree of attention required for processing visual stimuli (N2), and the degree of focal attention required for performing the task (P3). The vection-eliciting stimulation influenced the latency of P3 component and reaction times to targets in a similar fashion. Thus, our study reveals the electrophysiological underpinnings of the influence of vection-inducing visual stimulation on the performance of the simultaneous visual task.

However, it has to be acknowledged that our conclusions are limited in some respects. First, it is not ensured by the present findings that the ERP effects observed were due to the factual influence of vection. They could rather be explained by general visual stimulation of object motion that did not necessarily induce vection. This also refers to the behavioral result of reaction times to targets, which were shorter in the CS + PS condition than in the CM + PM condition. It can also be argued whether this effect was actually induced by the vection itself. Given that the mean proportions of subjectively felt sensations of vection were moderate (.30 in the CM + PM condition; .22 in the CS + PS condition), it is debatable whether participants actually perceived vection when making their responses. Likewise, it is possible that differences in reaction times to targets between static pattern and motion patterns were caused by general visual stimulation that did not induce vection. It is also possible that the motion of the central visual field interfered with the identification of the targets that were presented in that field and that this interference, not the vection itself, was the main reason of slowing down responses to targets in the oddball task. In an effort to disentangle these questions, we performed additional analysis on reaction times to targets that were presented before subjects’ button presses indicating that they subjectively felt the sensation of vection and after that, within the same motion pattern (CM + PM or CS + PM). The results of this analysis turned out to be significant, indicating that reaction times were slower after self-reported vection sensation than before it. This pattern of results was consistent with our predictions and reassured that it was factually the sensation of vection that slowed down reaction times to targets in the oddball task.

Lastly, the important limitation of our study is that the oddball task was very easy to perform which makes it difficult to really assess an effect of vection upon it. It is possible that our findings would have been different if we had used more difficult task. Thus, further studies are needed to determine the generalizability of the influence of vection-inducing stimulation (or, preferably, the influence of vection itself) to other, more complex, tasks.

## References

[CR1] Beer J, Blakemore C, Previc FH, Liotti M (2002). Areas of the human brain activated by ambient visual motion, indicating three kinds of self-movement. Exp Brain Res.

[CR2] Brandt T, Bartenstein P, Janek A, Dieterich M (1998). Reciprocal inhibitory visual-vestibular interaction. Visual motion stimulation deactivates the parieto-insular cortex. Brain.

[CR3] Comerchero MD, Polich J (1999). P3a and P3b from typical auditory and visual stimuli. Clin Neurophysiol.

[CR4] Delorme A, Sejnowski T, Makeig S (2007). Enhanced detection of artifacts in EEG data using higher-order statistics and independent component analysis. Neuroimage.

[CR5] Deutschländer A, Bense S, Stephan T, Schwaiger M, Dieterich M, Brandt T (2004). Rollvection versus linearvection: comparison of brain activations in PET. Hum Brain Mapp.

[CR6] Dichgans J, Brandt T, Held R, Leibowitz HW, Teuber HL (1978). Visual-vestibular interaction: effects on self-motion perception and postural control. Handbook of sensory physiology.

[CR7] Folstein JR, Van Petten C (2008). Influence of cognitive control and mismatch on the N2 component of the ERP: a review. Psychophysiology.

[CR8] Hillyard SA, Vogel EK, Luck SJ (1998). Sensory gain control (amplification) as a mechanism of selective attention: electrophysiological and neuroimaging evidence. Philos Trans R Soc B.

[CR9] Johansson G (1977). Studies on visual perception of locomotion. Perception.

[CR10] Kahneman D (1973). Attention and effort.

[CR11] Kennedy RS, Hettinger LJ, Harm DL, Ordy JM, Dunlap WP (1995). Psychophysical scaling of circular vection (CV) produced by optokinetic (OKN) motion: individual differences and effects of practice. J Vestib Res-Equil.

[CR12] Keshavarz B, Berti S (2014). Integration of sensory information precedes the sensation of vection: a combined behavioral and event-related brain potential (ERP) study. Behav Brain Res.

[CR13] Keshavarz B, Campos JL, Berti S (2015). Vection lies in the brain of the beholder: EEG parameters as an objective measurement of vection. Front Psychol.

[CR14] Kleinschmidt A, Thilo KV, Büchel C, Gresty MA, Bronstein AM, Frackowiak RS (2002). Neural correlates of visual-motion perception as object-or self-motion. Neuroimage.

[CR15] Koenderink JJ (1986). Optic flow. Vision Res.

[CR16] Kovács G, Raabe M, Greenlee MW (2008). Neural correlates of visually induced self-motion illusion in depth. Cereb Cortex.

[CR17] Lappe M, Bremmer F, Van den Berg AV (1999). Perception of self-motion from visual flow. Trends Cogn Sci.

[CR18] Lavie N (2005). Distracted and confused? Selective attention under load. Trends Cogn Sci.

[CR19] Lee DN (1980). The optic flow field: the foundation of vision. Philos Trans R Soc B.

[CR20] Luck SJ, Luck SJ, Kappenman ES (2012). Electrophysiological correlates of the focusing of attention within complex visual scenes: N2pc and related ERP components. The Oxford handbook of event-related potential components.

[CR21] Luck SJ (2014). An introduction to the event-related potential technique.

[CR22] Luck SJ, Kappenman ES, Luck SJ, Kappenman ES (2012). ERP components and selective attention. The Oxford handbook of event-related potential components.

[CR23] Marois R, Ivanoff J (2005). Capacity limits of information processing in the brain. Trends Cogn Sci.

[CR24] Ng VW, Eslinger PJ, Williams SC, Brammer MJ, Bullmore ET, Andrew CM, Suckling J, Morris RG, Benton AL (2000). Hemispheric preference in visuospatial processing: a complementary approach with fMRI and lesion studies. Hum Brain Mapp.

[CR25] Palmisano S, Allison RS, Schira MM, Barry RJ (2015). Future challenges for vection research: definitions, functional significance, measures, and neural bases. Front Psychol.

[CR26] Polich J (2007). Updating P300: an integrative theory of P3a and P3b. Clin Neurophysiol.

[CR27] Suwazono S, Machado L, Knight RT (2000). Predictive value of novel stimuli modifies visual event-related potentials and behavior. Clin Neurophysiol.

[CR28] Thilo KV, Kleinschmidt A, Gresty MA (2003). Perception of self-motion from peripheral optokinetic stimulation suppresses visual evoked responses to central stimuli. J Neurophysiol.

[CR29] Tokumaru O, Kaida K, Ashida H, Yoneda I, Tatsuno J (1999). EEG topographical analysis of spatial disorientation. Aviat Space Environ Med.

[CR30] Wiest G, Amorim MA, Mayer D, Schick S, Deecke L, Lang W (2001). Cortical responses to object-motion and visually-induced self-motion perception. Cogn Brain Res.

